# Homochiral BINOL-based macrocycles with π-electron-rich, electron-withdrawing or extended spacing units as receptors for C_60_

**DOI:** 10.3762/bjoc.10.132

**Published:** 2014-06-06

**Authors:** Marco Caricato, Silvia Díez González, Idoia Arandia Ariño, Dario Pasini

**Affiliations:** 1 Department of Chemistry, University of Pavia, Viale Taramelli 10, 27100 Pavia, Italy; 2 INSTM Research Unit, Department of Chemistry, University of Pavia, 27100 Pavia, Italy

**Keywords:** BINOL, C_60_, carbon nanomaterials, carbon nanostructures, chirality, macrocycles, sensors, supramolecular chemistry

## Abstract

The “one-pot” synthesis of several homochiral macrocycles has been achieved by using π-electron-rich, electron-deficient or extended aromatic dicarboxylic acids in combination with an axially-chiral dibenzylic alcohol, derived from enantiomerically-pure BINOL. Two series of cyclic adducts with average molecular *D*_2_ and *D*_3_ molecular symmetries, respectively, have been isolated in pure forms. Their yields and selectivities deviate substantially from statistical distributions. NMR and CD spectroscopic methods are efficient and functional in order to highlight the variability of shapes of the covalent macrocyclic frameworks. The larger *D*_3_ cyclic adducts exhibit recognition properties towards C_60_ in toluene solutions (up to log *K*_a_ = 3.2) with variable stoichiometries and variable intensities of the charge-tranfer band upon complexation.

## Introduction

Shape persistent macrocycles are carbon-based nanomaterials and are more and more in demand. They enrich the molecular toolkit available to a variety of disciplines, e.g., supramolecular chemistry and materials science [1–4]. Shape persistency properties are traditionally sought after for enhancing the recognition toward suitable guests [5–10], and for the self-assembly, of stable organic nanotubes from the macrocyclic structures as molecular building blocks [11]. Cyclic peptides [12–14], phenylacetylene macrocycles [15], amide-containing macrocycles [16], and urea-based structures [17] have all been exploited to develop the nanotube concept.

Efficient supramolecular receptors for C_60_ and higher fullerenes have been already reported in the literature and research in this area is still very active [18–19]. Jasti and co-workers have demonstrated how cyclo-*p*-phenylenes of suitable size are able to form very stable complexes with C_60_ [20]. Aida and co-workers have reported on π-electron rich, porphyrin-based cyclic structures, which are able to selectively recognize C_60_ [21]. In subsequent work, similar porphyrin-based systems have been made chiral by substituting one of the nitrogen atoms, and the enantioselective complexation of chiral higher fullerenes (C_84_) has been demonstrated [22]. Complexes of C_60_ and C_70_ with large, calix-type macrocycles formed by π-electron deficient pyridine aromatic rings bridged by a nitrogen heteroatom, have been reported and characterized in terms of thermodynamic stability by using fluorescence measurements [23]. Martin, Perez et al. have reported on a series of extended TTF units able to form strong complexes with C_60_ and C_70_ in organic solutions [24–25].

We have recently reported an efficient protocol for the preparation of several chiral macrocycles incorporating BINOL (1,1′-bi-2-naphthol) units, through the formation of bridging ester functionalities, which ensure chemical inertness for the purposes of supramolecular sensing, recognition, or self-assembly. We have shown their application in the chiroptical sensing of organic or ionic species [26–39]. We have previously reported how some of our systems are able to sense C_60_ in toluene solutions, and how the recognition behavior is shape selective [28]. In the case of extended [2 + 2] macrocycles, induced CD activity in the characteristic UV–vis absorption bands of C_60_ showed how the chirality of the macrocyclic units could be transferred to the overall supramolecular ensemble [30].

In this paper, we extend on our previous studies by describing the synthesis of optically-active *D*_2_ and *D*_3_ macrocycles, whose spacing units are systematically changed in terms of their electronic nature, and we report on the recognition behavior towards C_60_.

## Results and Discussion

### Design, synthesis and spectroscopic characterization

**Scheme 1:**
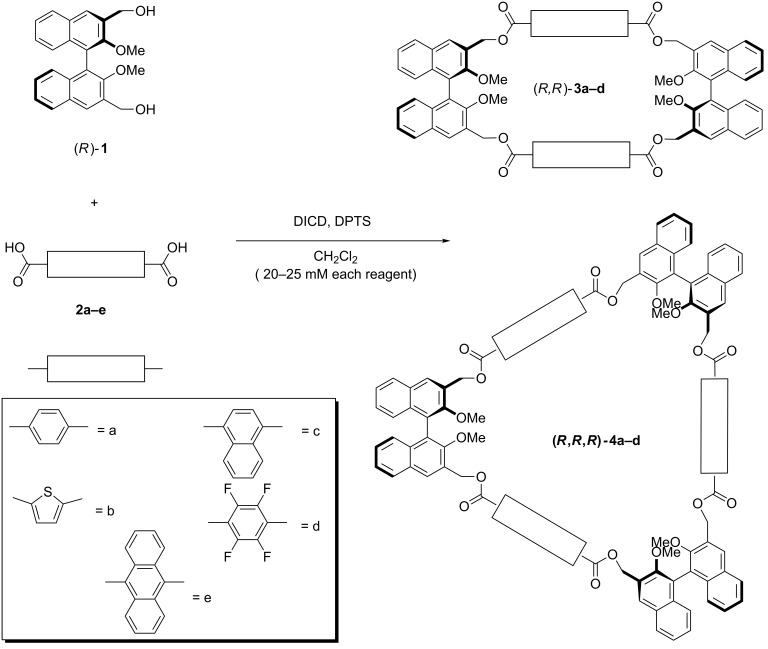
Synthesis of macrocycles **3** and **4**.

In our design approach, rigid, aromatic dicarboxylic acid spacers are combined with a BINOL derivative with masked phenol functionalities in the 2,2’-positions and benzylic alcohol functionalities in the 3,3’-positions (diol **1** in Scheme 1). The incorporation of sp^3^ methylene carbon atoms in the cyclic structure adds a certain degree of conformational freedom to the covalent structure. This feature balances the distortion of the planarity which is inevitably introduced by the binaphthyl units. Enantiopure (*R*)-**1** [40] was used in all cases, in order to achieve homochiral macrocycles. As previously reported in the case of terephthalic acid, both the [2 + 2] and the [3 + 3] macrocycles (compounds **3** and **4** in Scheme 1) were obtained.

The compounds have average molecular *D*_2_ and *D*_3_ point group symmetries, respectively. Our optimized esterification reaction protocol is carried out at intermediate dilution levels (each reagent 20–25 mM) in CH_2_Cl_2_. Several dicarboxylic acids with varying electronic structures and steric demands were tested. In the case of 9,10-anthracenedicarboxylic acid (**2e**), no cyclic product could be obtained, and only oligomeric, baseline materials were detected. Isolated yields, after column chromatography, are reported in Table 1. The yields and selectivities of isolated products are unusual, considering that the [3 + 3] macrocycles are sometimes formed with similar synthetic efficiency as the [2 + 2] macrocycles (entries 2 and 3 in Table 1). It is very likely that those conformational preferences dominate in this context.

**Table 1:** Yields of isolated cyclized products.^a^

Entry	Diacid precursor	Macrocyle **3**	Macrocycle **4**

1^b^	**2a**	18	9
2	**2b**	6	4
3	**2c**	8	5
4	**2d**	18	4
5	**2e**	0	0

^a^Isolated yield after colum chromatography. For conditions, see experimental. ^b^Data taken from ref. [28].

**Figure 1:**
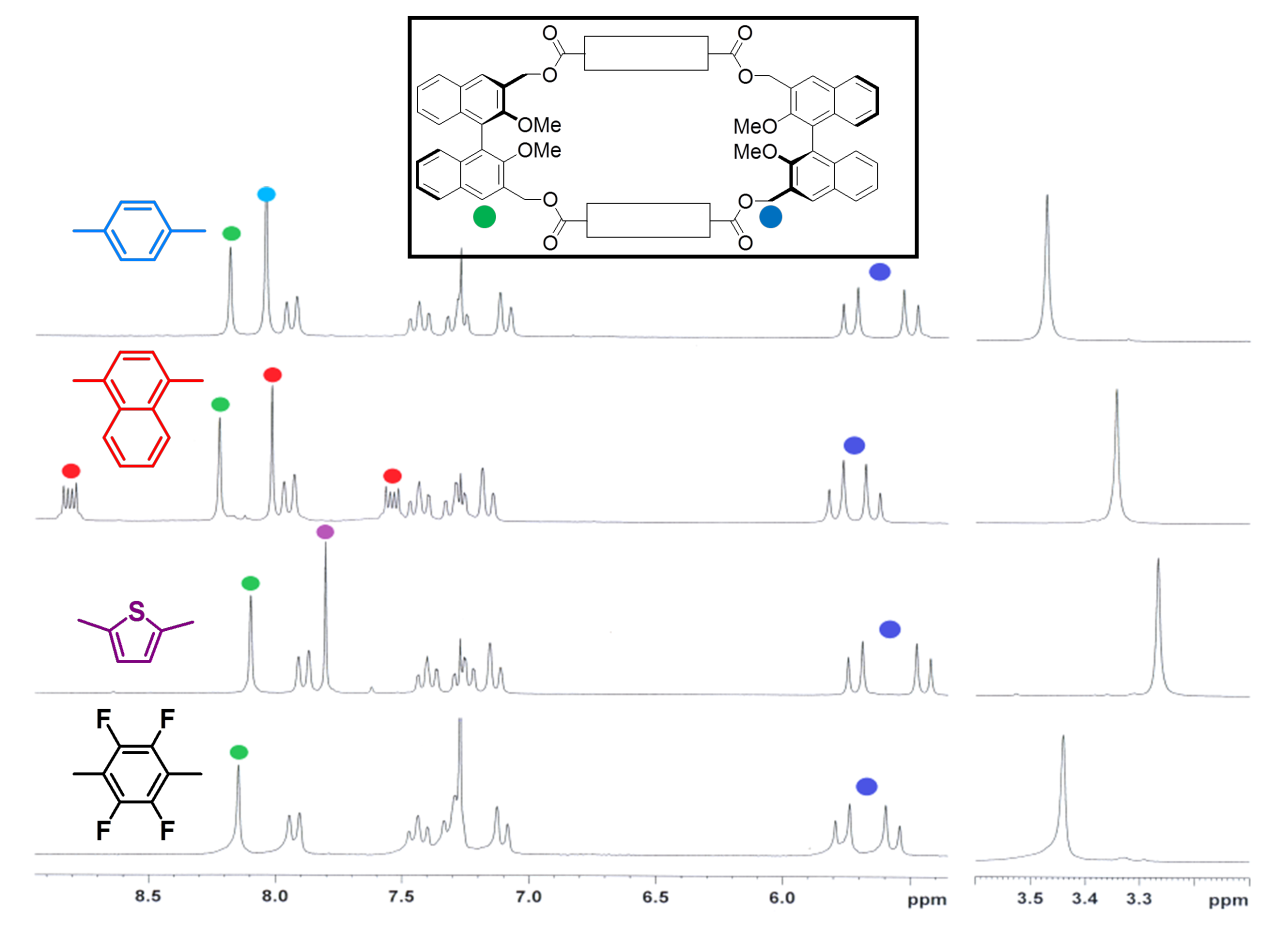
^1^H NMR spectra of macrocycles **3a–d**, with key proton resonances for the spacing units and key benzylic and BINOL-based units highlighted with different colors.

**Figure 2:**
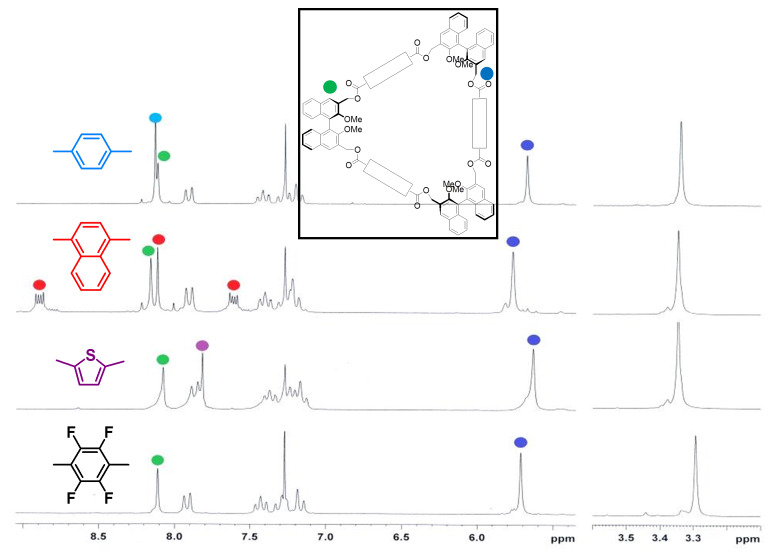
^1^H NMR spectroscopy of macrocycles **4a–d**, with proton resonances for the spacing units and key benzylic and BINOL-based units highlighted with color.

The macrocycles were correctly identified by NMR spectroscopy and mass spectrometry (see Supporting Information File 1). The room temperature ^1^H NMR spectra for all cyclic compounds display only one set of signals for each group of symmetry-related proton resonances, showing that all dynamic processes are fast on the NMR timescale at this temperature (Figure 1 and Figure 2).

In the case of macrocycles **3**, significant differences in the chemical shift of the proton resonances of the methoxy groups (Table 2, from 3.27 ppm to 3.48 ppm) and of the BINOL H-4,4‘ proton resonances (from 8.02 ppm to 8.19 ppm) could be detected. These significant differences tend to cancel out in the case of the larger macrocycles **4**, pointing to a more flexible nature of the latter class of macrocycles. In the case of all the *D*_3_ macrocycles **4**, the CH_2_ benzylic proton resonances appear as collapsed AB systems at room temperature (Figure 2). They demonstrate a peculiar arrangement for the two diastereotopic methylene protons, substantially different from that of the more rigid *D*_2_ symmetrical analogues **3**, in which the methylene proton resonances appear as well-defined AB systems. Small amounts (5–10%) of impurities in macrocycles **4** were difficult to remove by flash column chromatography. These byproducts were tentatively characterized as higher oligomers from their NMR pattern.

**Table 2:** Selected chemical shifts for compounds in CDCl_3_ (25 °C).^a^

Entry	Compound	Binol-H4,4'^b^	Benzylic CH_2_	OCH_3_

1^c^	**3a**	8.19	5.63^d^	3.48
2	**3b**	8.10	5.60^d^	3.27
4	**3c**	8.02	5.72^d^	3.34
5	**3d**	8.15	5.67^d^	3.44
6^c^	**4a**	8.15	5.68^e^	3.35
7	**4b**	8.10	5.63^e^	3.31
8	**4c**	8.13	5.79^e^	3.35
9	**4d**	8.11	5.71^e^	3.29

^a^All spectra recorded at 5–10 mM sample concentration. ^b^Resonances related to the singlet corresponding to the proton in the 4,4'-positions of the BINOL skeleton. ^c^Data taken from ref. [28]. ^d^Multiplicity of the ^1^H NMR signal: Quartet, AB system. ^e^Multiplicity of the ^1^H NMR signal: collapsed AB system.

**Figure 3:**
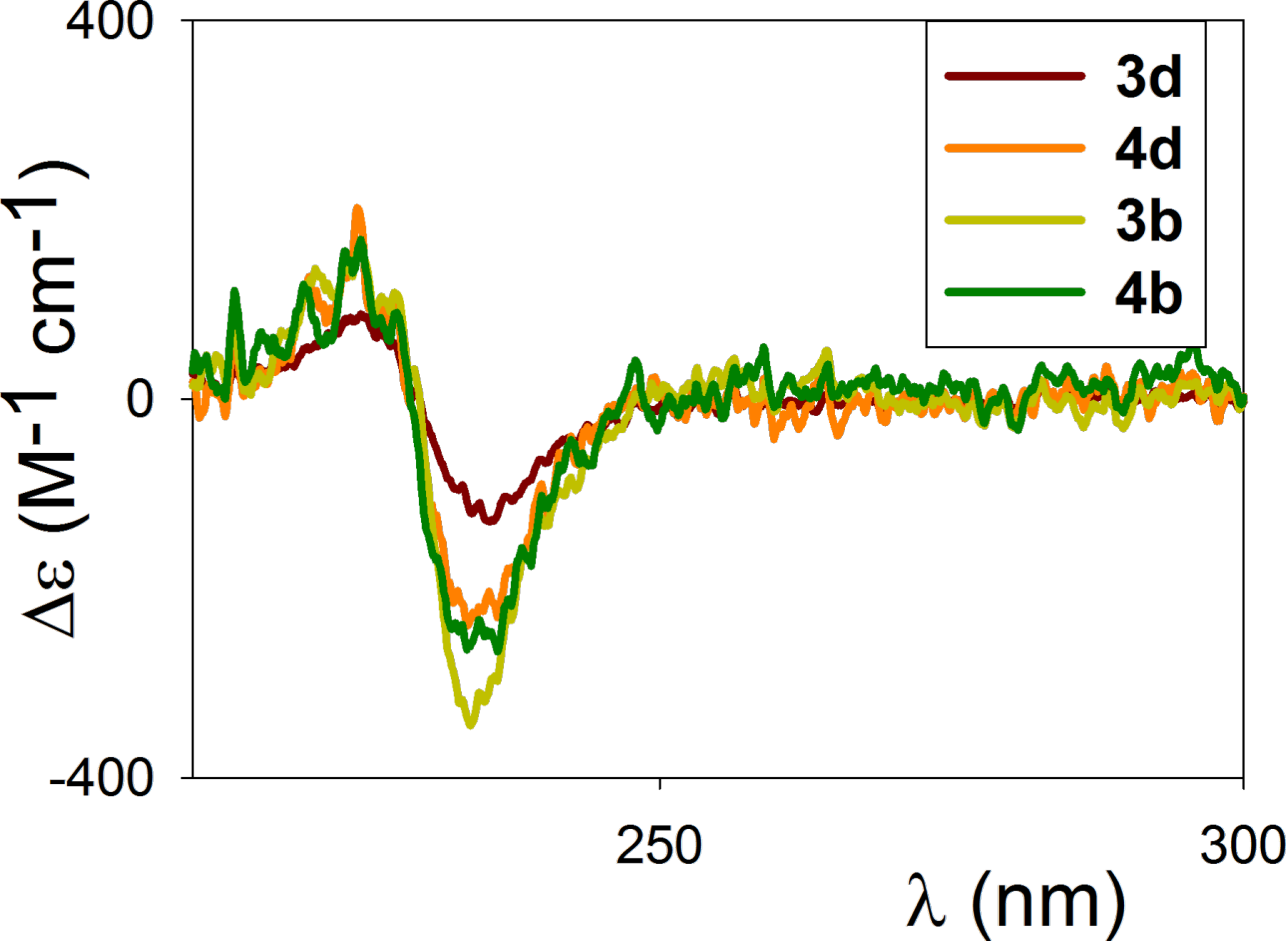
CD spectra of macrocycles **3b**, **3d**, **4b**, **4d** in EtOH (0.5–12 × 10^−6^ M).

The UV–vis absorption spectra in EtOH of a selection of macrocyclic compounds (**3b**, **3d**, **4b** and **4d** with π-electron rich and π-electron deficient spacing units, Figure S1, Supporting Information File 1) show the major absorption band centered around 230 nm, typical of the binaphthyl chromophore [41]. Circular dichroism spectroscopy of the same macrocycles show the exciton couplet typical of binaphthyl moieties (Figure 3), corresponding to the maximum absorption band in the UV–vis spectra.

Induced CD activity associated with other absorption bands in the UV–vis spectra (particularly intense in the case of **3b** and **4b**, Figure S1, Supporting Information File 1) could not be detected. The intensity of the low energy component of the couplet is significantly different in the case of the smallest macrocycles (Δε values of −113 for **3d** and −326 for **3b**). For substituted 2,2'-binaphthol derivatives, it has been reported that the low energy component values are related to variations of the dihedral angle between the naphthyl units due to the steric hindrance of the substituents in the 2,2'-positions [41]. Since compounds **3** possess the same substituent (OMe) in the 2,2'-positions, the variability of these values and thus the variation of the dihedral angle of the binaphthyl units, must depend on the buttressing effects of the neighbouring 3,3’-benzylic ester positions. They, in turn, can derive from the differing macrocyclic structural flexibility or from an equally rigid conformation altering the coupling of the benzylic protons. These data corroborate the NMR data in highlighting that the influence of the differing geometrical shapes of the spacing units on the macrocyclic conformation is strong in the case of the more rigid [2 + 2] macrocycles **3**, but it tends to cancel out in the case of the more flexible [3 + 3] macrocycles **4**.

### Complexation studies

**Figure 4:**
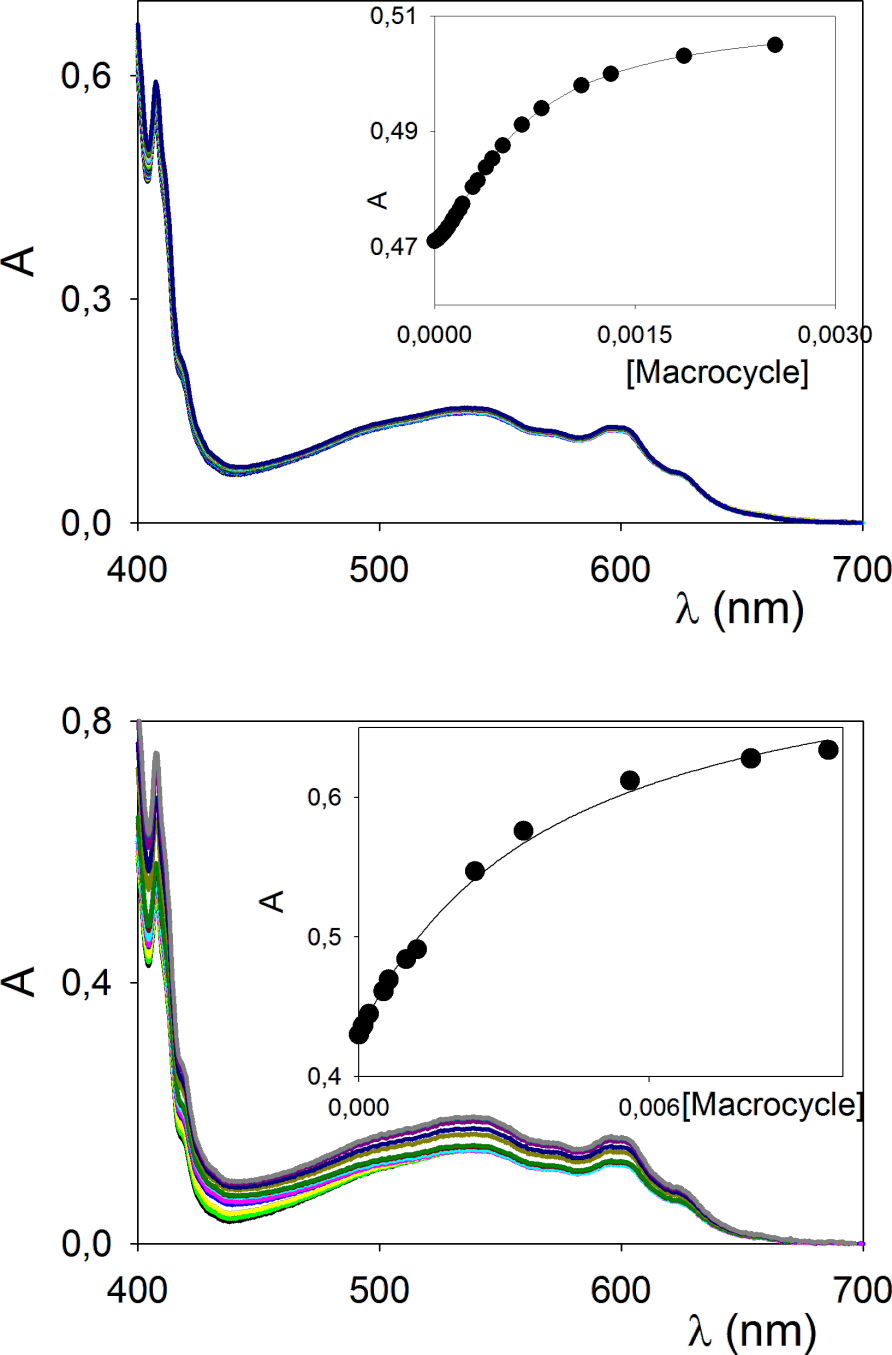
UV–vis titration of C_60_ (1.8 × 10^−4^ M) in toluene with increasing amounts of macrocycle **4b** (top) and **4d** (bottom). Inset: titration profiles at 405 nm, and relative best fitting curves obtained with the Hill equation (top) or a 1:1 binding equation (bottom).

Titrations of a solution of C_60_ with increasing amounts of macrocycles **3b–d** in toluene solutions resulted in no detectable changes in the UV–vis spectra, similarly to what was previously described for the [2 + 2] adduct **3a**. In the case of macrocyles **4b** and **4d**, instead, an enhancement of the absorption band above 400 nm could be readily detected (Figure 4). This behavior is similar to previously reported cases in terms of band shape, involving both cyclic π-electron rich and π-electron deficient substrates [23,25].

The calculated thermodynamic binding constants are reported in Table 3. In the case of macrocycle **4d**, a 1:1 binding isotherm could be efficiently employed to fit the titration data, strongly indicating that a 1:1 binding behavior between the host and the C_60_ guest is predominant in solution. The insertion of the π-electron deficient tetrafluoroterephthalic moieties lowers the affinity with the electron acceptor C_60_ guest, when compared with the reference host **4a** (Table 3, entry 1 vs entry 4). This is reasonable as the C_60_ core is an acceptor of electron density, so that π-electron neutral or rich substrates are more suitable for complexation.

**Table 3:** Thermodynamic binding constants of the complexation of C_60_ with macrocycles **4** in toluene (25 °C).^a^

Entry	Compound	*k*_a_^b^	Hill coefficient^c^

1^c^	**4a**	1100 ± 100	1
2	**4b**	1600 ± 200	1.5
3	**4c**	No binding	–
4	**4d**	200 ± 40	1

^a^C_60_ concentration constant at 1.8 × 10^−4^ M in all cases. The *k*_a_ values are the average of two independent tirations. ^b^In M^−1^. ^c^Data taken from ref. [28].

In the case of **4b**, an acceptable fitting with the 1:1 binding equation could not be achieved, indicating multiple binding stoichiometries in solution. Data treatment with the Hill equation [42] gave an average binding constant of 1600 M^−1^, thus confirming a higher affinity for the π-electron rich thiophene-derived spacer units. The Hill coefficient [43–44] suggests the presence of concomitant 1:1 and 1:2 C_60_:macrocycle complexes in solution.

Even more interestingly, the titration profiles in the insets of Figure 4 display marked differences for the calculated molar absorbivity values of the **4b**@C_60_ and **4d**@C_60_ complexes at saturation (2800 and 4000 M^−1^ cm^−1^, respectively). In the case of **4c**, no variation in the UV–vis spectra was detected. It is likely that in this case the steric demand of the naphthalene spacing units does not allow for the optimal positioning of the C_60_ and the formation of a **4c**@C_60_ complex of measurable stability.

## Conclusion

We have reported on the synthesis and characterization of novel homochiral macrocycles, built upon resolved 1,1’-binaphthyl scaffolds, which incorporate either π-electron rich, π-electron deficient or π-extended spacing units. The cyclic adducts are obtained in an acceptable yield in a one-pot synthetic procedure, and easily purified by flash column chromatography. NMR and CD spectroscopy give an insight into the conformational properties of these cyclic structures and indicate a more rigid structure for the [2 + 2] adducts, whereas the [3 + 3] adducts are more flexible. The latter are also capable of binding C_60_ in toluene solutions. Both the thermodynamic strengths and the optical absorptivity coefficients of the complexes in solution give insights into the role of nonspecific host–guest interactions (such as π–π stacking) for the overall stabilization of the complexes. We are currently designing binaphthyl-based hosts for the enantioselective recognition and separation of higher fullerenes and chiral nanotubes. These binaphthyl-based hosts may also be utilized as chiroptical sensors for chiral carbon-based nanomaterials in functional nanochemical environments.

## Experimental

**General experimental.** All commercially available compounds were purchased from commercial sources and used as received. Compounds (*R*)-**1** [40], **3a** [28], **4a** [28] and *p*-toluenesulfonic acid 4-dimethylaminopyridinium salt (PTSA-DMAP) [45] were prepared according to literature procedures. THF (Na, benzophenone) and CH_2_Cl_2_ (CaH_2_) were dried before use. Analytical thin-layer chromatography was performed on silica gel, chromophore loaded, and with commercially available plates. Flash chromatography was carried out by using silica gel (pore size 60 Å, 230–400 mesh). ^1^H and ^13^C NMR spectra were recorded from solutions in CDCl_3_ on 200 or 300 MHz spectrometer with the solvent residual proton signal or tetramethylsilane as a standard. The UV–vis spectroscopic studies were recorded by means of commercially-available spectrophotometers. Mass spectra were recorded by using an electrospray ionization instrument. Optical rotations were measured on a polarimeter with a sodium lamp (λ = 589 nm) and are reported as follows: [α]_D_^rt^ (*c* = g (100 mL solvent)^−1^). CD spectroscopy was performed with a spectropolarimeter; spectra were recorded at 25 °C at a scanning speed of 50 nm min^−1^ and were background corrected. The reported spectra are the instrument-averaged results of four consecutive scans.

**General procedure for the preparation of macrocycles 3 and 4.** In a manner similar to the procedure described in [28], a solution of DICD (diisopropylcarbodiimide, 3 equivalents vs diol and dicarboxylic acid) in a minimum amount of dry CH_2_Cl_2_ is added to a solution of the appropriate dicarboxylic acid (20–25 mM), (*R*)*-***1** (20–25 mM), PTSA-DMAP (2 equivalents) in dry CH_2_Cl_2_ under stirring and N_2_. The solution is stirred overnight, and then H_2_O (10 mL) is added. The aqueous phase is extracted with CH_2_Cl_2_ (3×), the organic phase is washed with H_2_O (3×) and dried (MgSO_4_). The products are then purified by flash chromatography.

Macrocycles (*R*,*R*)*-***3b** and (*R*,*R*,*R*)-**4b***.* From 2,5-thiophenedicarboxylic acid (115 mg, 0.67 mmol, 1 equiv), DICD (312 µL, 2.01 mmol, 3 equiv), (*R*)-**1** (250 mg, 0.67 mmol, 1 equiv), PTSA-DMAP (414 mg, 1.337 mmol, 2 equiv). Purified by flash column chromatography (hexanes/EtOAc 8:2 to 7:3) to yield **3b** (21 mg, 6%) and **4b** (13 mg, 4%) as white solids. (*R*,*R*)**-3b**. [α]_D_^25^ +54 (*c* 0.0015, CH_2_Cl_2_); ESIMS *m*/*z* (%): 1043 ([M + Na]^+^, 100); ^1^H NMR (CDCl_3_, 200 MHz, 25 °C) δ 8.10 (s, 4H, binaphthyl), 7.89 (d, 4H, binaphthyl), 7.81 (s, 4H, thiophene), 7.38 (t, 4H, binaphthyl), 7.27 (m, 4H, binaphthyl), 7.16 (d, 4H, binaphthyl), 5.60 (dd, 8H, CH_2_), 3.27 (s, 12H, -OCH_3_); ^13^C NMR (CDCl_3_, 75 MHz, 25 °C) δ 161.3 (Cq), 155.9 (Cq), 138.2 (Cq), 134.6 (Cq), 133.5 (CH), 132.9 (CH), 130.0 (Cq), 128.3 (CH), 128.2 (Cq), 127.1 (CH), 125.5 (CH), 125.0 (CH), 124.2 (Cq), 64.2 (CH_2_), 61.4 (CH_3_). (*R*,*R*,*R*)**-4b**. [α]_D_^25^ +43 (*c* 0.0015, CH_2_Cl_2_); ESIMS *m*/*z* (%): 1553 ([M + Na]^+^, 100); ^1^H NMR (CDCl_3_, 200 MHz, 25 °C) δ 8.10 (s, 6H, binaphthyl), 7.95–7.80 (m, 12H, binaphthyl + thiophene), 7.35 (t, 6H, binaphthyl), 7.5–7.1 (m, 12H, binaphthyl), 5.63 (s, 12H, CH_2_), 3.31 (s, 18H, OCH_3_); ^13^C NMR (CDCl_3_, 75 MHz, 25 °C) δ 161.2 (Cq), 154.9 (Cq), 138.7 (Cq), 134.3 (Cq), 133.3 (CH), 130.8 (CH), 130.1 (Cq), 128.5 (Cq), 128.1 (CH), 126.9 (CH), 125.5 (CH), 125.1 (CH), 124.4 (Cq), 63.6 (CH_2_), 61.3 (CH_3_).

Macrocycles (*R*,*R*)-**3c** and (*R*,*R*,*R*)-**4c***.* From 1,4-naphthalenedicarboxylic acid (144 mg, 0.67 mmol, 1 equiv), DICD (312 µL, 2.0 mmol, 3 equiv), (*R*)-**1** (250 mg, 0.67 mmol, 1 equiv), PTSA-DMAP (414 mg, 1.34 mmol, 2 equiv). Purified by flash column chromatography (hexanes/EtOAc 7:3) to yield **3c** (30 mg, 8%) and **4b** (17 mg, 5%) as white solids. (*R*,*R*)**-3c**. [α]_D_^25^ +97 (*c* 0.0015, CH_2_Cl_2_); ESIMS *m*/*z* (%): 1131 ([M + Na]^+^, 100); ^1^H NMR (CDCl_3_, 200 MHz, 25 °C) δ 8.80 (m, 4H, naphthalene), 8.22 (s, 4H, naphthalene), 8.02 (s, 4H, binaphthyl), 7.94 (d, 4H, binaphthyl), 7.53 (m, 4H, naphthalene), 7.43 (t, 4H, binaphthyl), 7.27 (m, 4H, binaphthyl), 7.16 (d, 4H, binaphthyl), 5.72 (q, 8H, CH_2_), 3.34 (s, 12H, OCH_3_); ^13^C NMR (CDCl_3_, 75 MHz, 25 °C) δ 167.0 (Cq), 155.8 (Cq), 134.8 (Cq), 133.1 (CH), 131.9 (Cq), 131.2 (Cq), 130.1 (Cq), 128.6 (Cq), 128.3 (CH), 127.9 (Cq), 127.7 (CH), 127.6 (CH), 127.1 (CH), 125.8 (CH), 125.5 (CH), 125.1 (CH), 124.4 (Cq), 64.1 (CH_2_), 61.3 (CH_3_). (*R*,*R*,*R*)**-4c**. [α]_D_^25^ +80 (*c* 0.0015, CH_2_Cl_2_). ESIMS *m*/*z* (%): 1687 ([M + Na]^+^, 100); ^1^H NMR (CDCl_3_, 200 MHz, 25 °C) δ 8.90 (m, 6H, naphthalene), 8.19 (s, 6H, naphthalene), 8.13 (s, 6H, binaphthyl); 7.90 (d, 6H, binaphthyl), 7.61 (m, 6H, naphthalene), 7.38 (t, 6H, binaphthyl), 7.20 (m, 12H, binaphthyl), 5.79 (s, 12H, CH_2_), 3.35 (s, 18H, OCH_3_); ^13^C NMR (CDCl_3_, 75 MHz, 25 °C) δ 166.8 (Cq), 155.1 (Cq), 134.4 (Cq), 131.7 (Cq), 131.4 (Cq), 131.0 (CH), 130.2 (Cq), 128.9 (Cq), 128.2 (CH), 127.9 (CH), 127.7 (CH), 126.9 (CH), 125.9 (CH), 125.5 (CH), 125.1 (CH), 124.3 (Cq), 63.6 (CH_2_), 61.2 (CH_3_).

Macrocycles (*R*,*R*)-**3d** and (*R*,*R*,*R*)-**4d***.* From tetrafluoroterephthalic acid (162 mg, 0.68 mmol, 1 equiv), DICD (317 µL, 2.04 mmol, 3 equiv), (*R*)-**1** (255 mg, 0.68 mmol, 1 equiv), PTSA-DMAP (423 mg, 1.36 mmol, 2 equiv). Purified by column chromatography (hexanes/EtOAc 8:2) to yield **3d** (71 mg, 18%), **4d** (10 mg, 4%) and a noticeable quantity of a higher cyclic adduct (8 mg), as white solids. (*R*,*R*)**-3d**. [α]_D_^25^ +8.3 (*c* 0.005, CH_2_Cl_2_); ESIMS *m*/*z* (%): 1176 ([M + Na]^+^, 100); ^1^H NMR (CDCl_3_, 200 MHz, 25 °C) δ 8.15 (s, 4H, binaphthyl), 7.93 (d, 4H, binaphthyl), 7.43 (t, 4H, binaphthyl), 7.27 (s, 4H, binaphthyl), 7.11 (d, 4H, binaphthyl), 5.67 (dd, 8H, CH_2_), 3.44 (s, 12H, -OCH_3_); ^13^C NMR (CDCl_3_, 75 MHz) δ 158.7 (Cq), 155.4 (Cq), 144.5 (Cq, dm, *J* = 255 Hz), 134.9 (Cq), 133.4 (CH), 130.0 (Cq), 128.3 (CH), 127.4 (CH), 127.3 (Cq), 125.4 (CH), 125.4 (Cq), 125.2 (CH), 118.0 (Cq, m), 65.4 (CH_2_), 61.1 (CH_3_). (*R*,*R*,*R*)**-4d**. [α]_D_^25^ +4.8 (*c* 0.005, CH_2_Cl_2_); ESIMS *m*/*z* (%): 1752 ([M + Na]^+^, 100); ^1^H NMR (CDCl_3_, 200 MHz, 25°C) δ 8.11 (s, 6H, binaphthyl), 7.92 (d, 6H, binaphthyl), 7.43 (t, 6H, binaphthyl), 7.27 (m, 6H, binaphthyl), 7.17 (d, 6H, binaphthyl), 5.71 (s, 12H, CH_2_), 3.29 (s, 18H, -OCH_3_); ^13^C NMR (CDCl_3_, 75 MHz) δ 158.7 (Cq), 154.9 (Cq), 144.3 (Cq, *J* = 263 Hz), 134.6 (Cq), 131.5 (CH), 130.1 (Cq), 128.3 (CH), 127.6 (CH), 127.2 (Cq), 125.5 (CH), 125.2 (CH), 124.1 (Cq), 117.6 (Cq, m), 64.9 (CH_2_), 61.0 (CH_3_).

**UV–vis titrations.** As described in [28], the titration experiments were conducted as follows: to a stock solution of C_60_ (solution A) in toluene were added several aliquots of the host (solution B) in toluene. Solution B is formed by the ligand at a higher concentration dissolved in solution A, so that the guest always remains at the same, constant concentration. In the case of a 1:1 binding isotherm (Figure 4, bottom), by employing a nonlinear fitting curve program, the plot of A against the macrocycle concentration x was fitted by Equation 1, thus affording the value of the association constant *K*_a_ and of the molar absorptivity of the complex ε_c_:

[1]
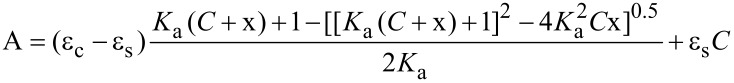


where A is the measured absorbance, x is the total concentration of titrant added, ε_c_ is the molar absorptivity of the complex, ε_s_ is the molar absorbivity of the substrate at the desired wavelength, which could be directly determined, *C* is the total concentration of the titrate (which is a constant quantity), and *K*_a_ is the association constant for the 1:1 complex [28].

The data for titrations of **4b** with C_60_ (Figure 4, top) were fitted to a general form of the Hill equation:


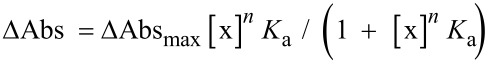


which can be conveniently rewritten in:

[2]
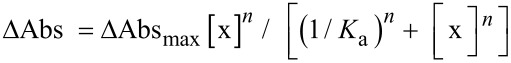


Equation 2 could be fitted employing a nonlinear fitting program according to the general equation: f(x) = a·x^b^/(c^b^ + x^b^), obtaining values of a = ΔAbs_max_, b = n (the Hill coefficient), c = 1/*K*_a_.

## Supporting Information

File 1 UV spectra for selected macrocycles, additional NMR and MS spectra for all newly synthesized macrocyles.
