# Supplementary material for: Homochiral BINOL-based macrocycles with π-electron-rich, electron-withdrawing or extended spacing units as receptors for C60
Source: Beilstein J Org Chem. 2014 Jun 6;10:1308–16. doi: 10.3762/bjoc.10.132 (PMC4077419; doi:10.3762/bjoc.10.132)
Supplement: File 1 — UV spectra for selected macrocycles, additional NMR and MS spectra for all newly synthesized macrocyles. [file Beilstein_J_Org_Chem-10-1308-s001.pdf]

Supporting Information

for

Homochiral BINOL-based macrocycles with π-electron-rich, electron-withdrawing or extended spacing units as receptors for C_{60}

Marco Caricato[1], Silvia Díez González[1], Idoia Arandia[1] and Dario Pasini*[,1,2]

Address: [1]Department of Chemistry, University of Pavia, Viale Taramelli 10, 27100 Pavia, Italy and [2]INSTM Research Unit, Department of Chemistry, University of Pavia, 27100 Pavia, Italy

Email: Dario Pasini - dario.pasini@unipv.it

* Corresponding author

UV spectra for selected macrocycles, additional NMR and MS spectra for all newly synthesized macrocyles

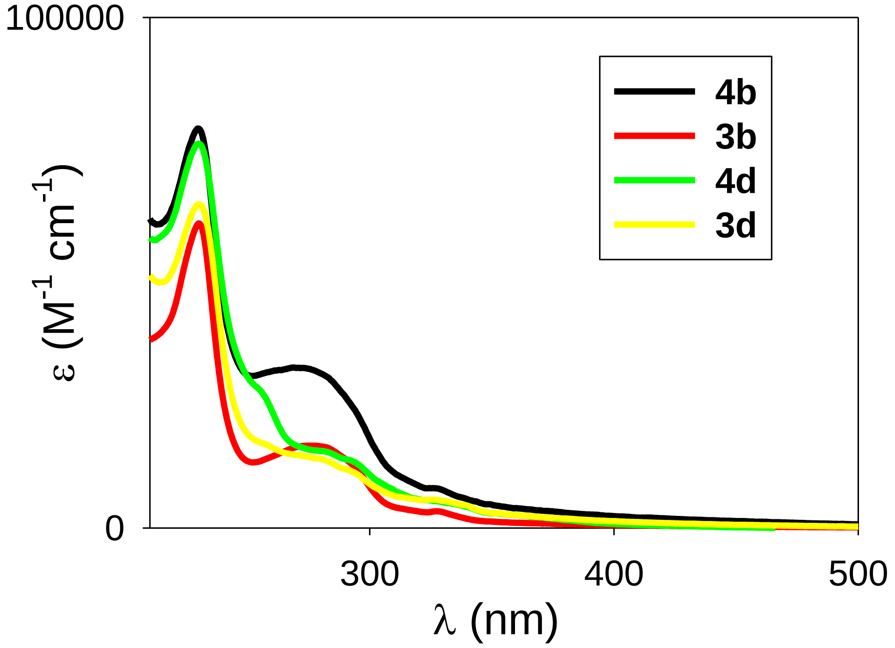

Figure S1: UV–vis spectra of macrocycles **3a**, **3d**, **4a** and **4d** in EtOH (0.5–12 × 10^{-6} M).

Compound (*R,R*)-**3b**.

^{13}C NMR ($CDCl_3$, 75 MHz)

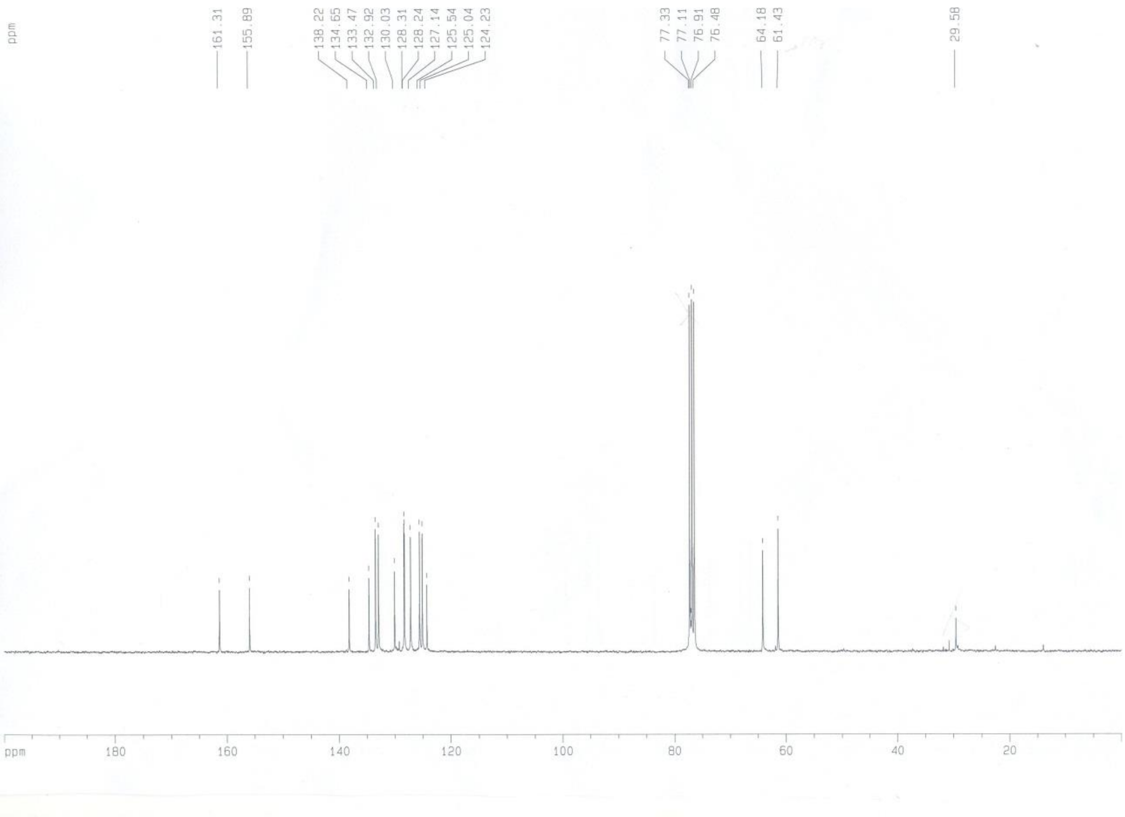

^{13}C NMR DEPT ($CDCl_3$, 75 MHz)

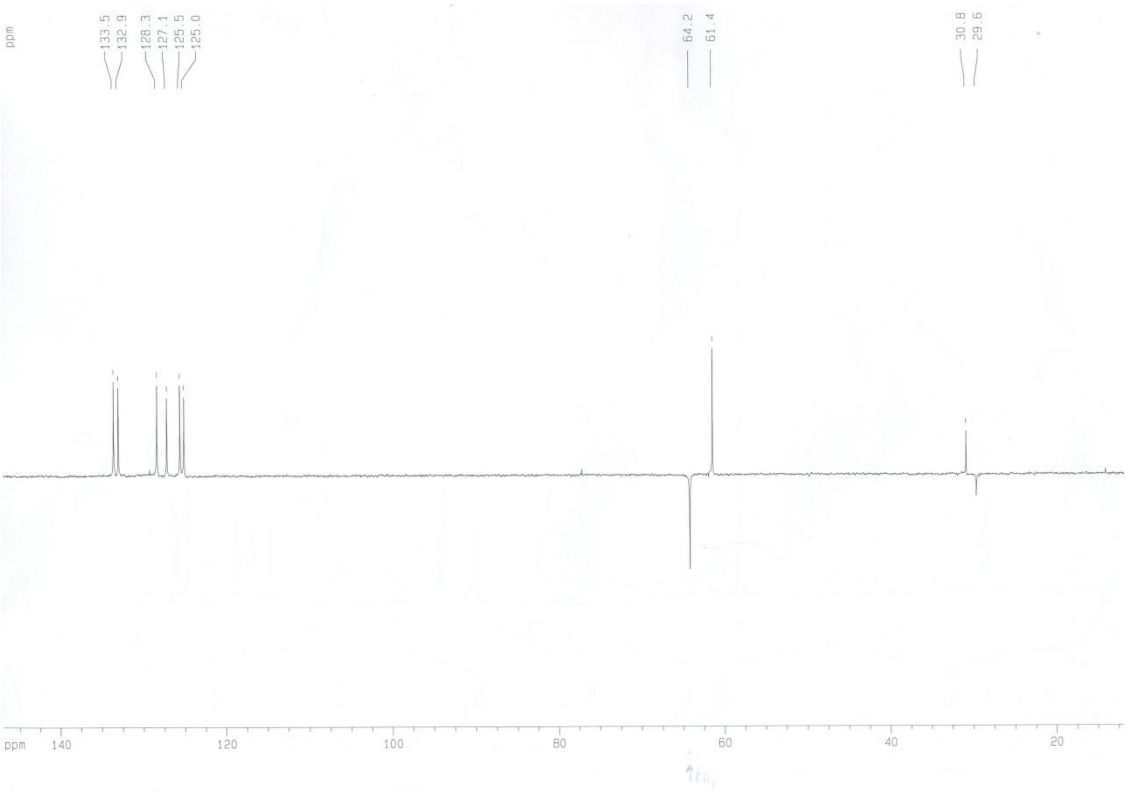

ESIMS

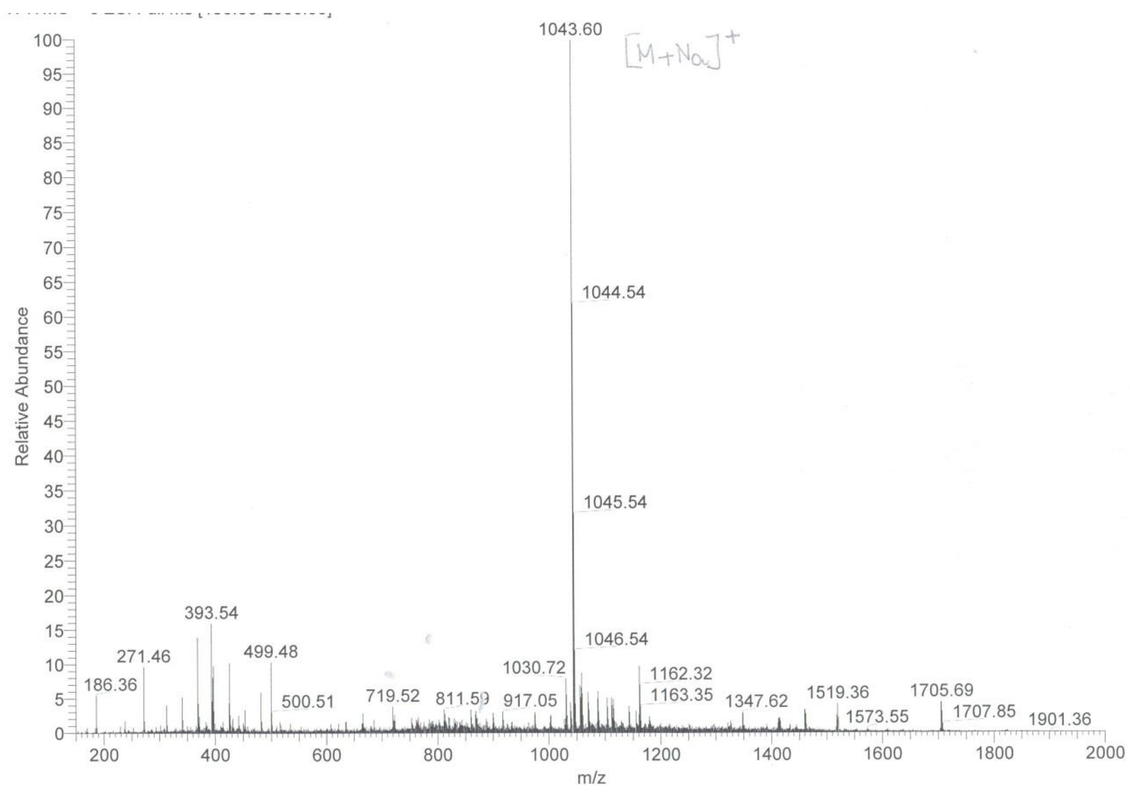

1043.60
[M+Na]+
1044.54
1045.54
1046.54
393.54
271.46
499.48
186.36
500.51
719.52
811.59
917.05
1030.72
1162.32
1163.35
1347.62
1519.36
1573.55
1705.69
1707.85
1901.36
Relative Abundance
m/z

Compound (*R,R*)-**3c***.*

^{13}C NMR ($CDCl_3$, 75 MHz)

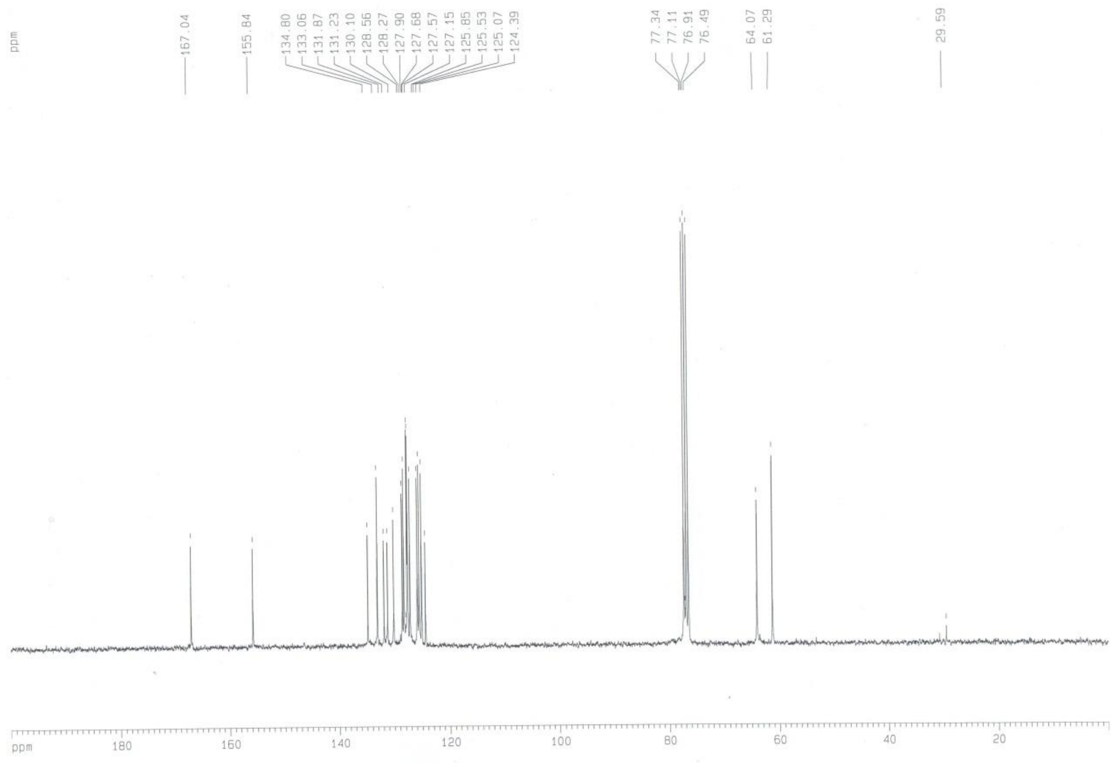

^{13}C NMR DEPT ($CDCl_3$, 75 MHz)

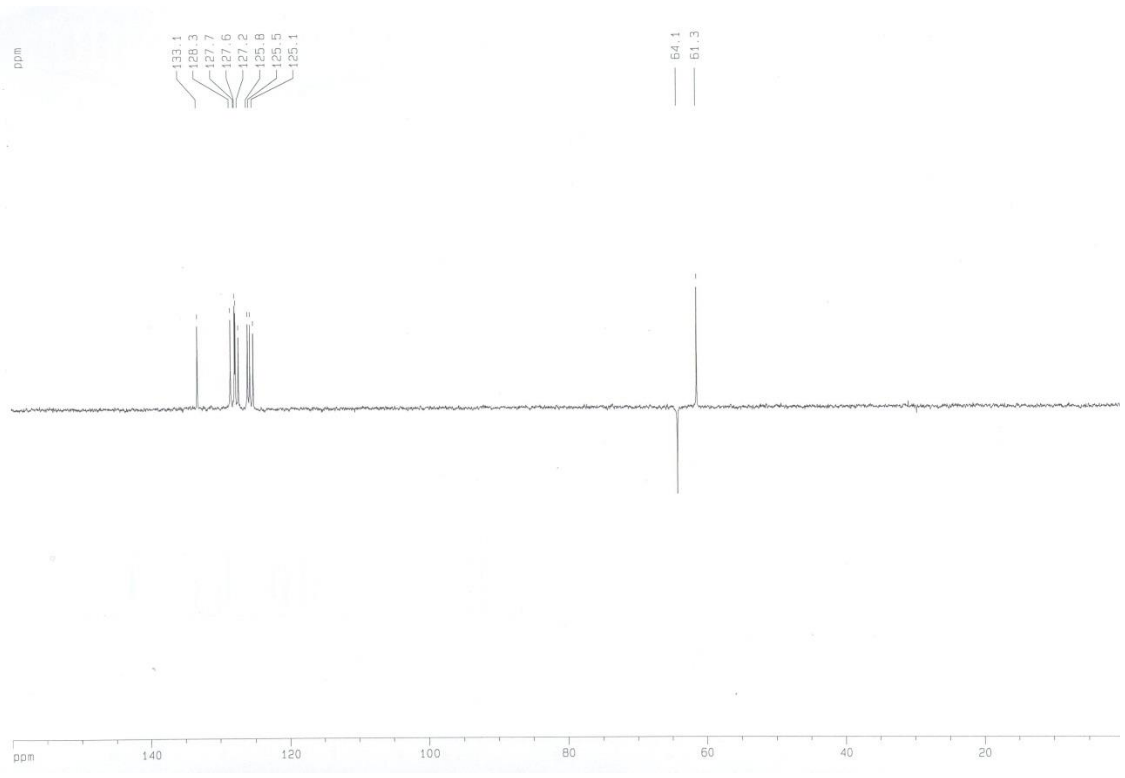

ESIMS

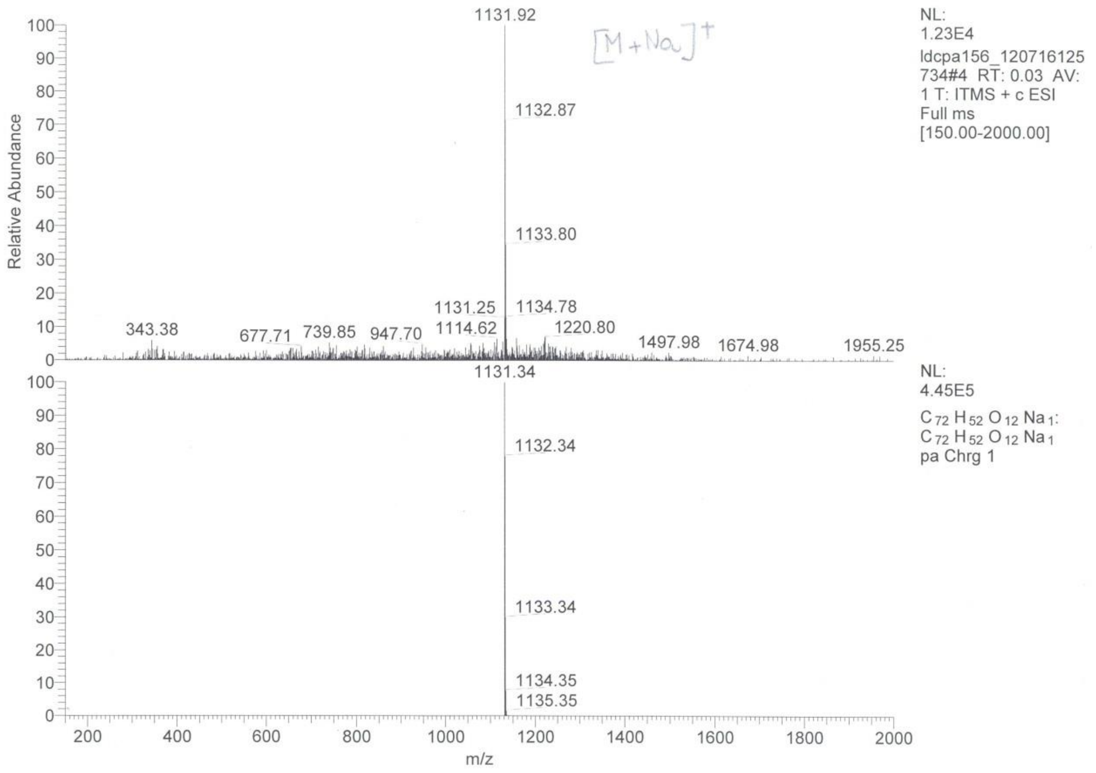

1131.92
[M+Na]+
NL:
1.23E4
ldcpa156_120716125
734#4 RT: 0.03 AV:
1 T: ITMS + c ESI
Full ms
[150.00-2000.00]
1132.87
1133.80
1131.25
1134.78
1114.62
1220.80
343.38
677.71
739.85
947.70
1497.98
1674.98
1955.25
Relative Abundance
1131.34
NL:
4.45E5
C 72 H 52 O 12 Na 1:
C 72 H 52 O 12 Na 1
pa Chrg 1
1132.34
1133.34
1134.35
1135.35
m/z

Compound (*R*,*R*)-**3d**

^{13}C NMR ($CDCl_3$, 75 MHz)

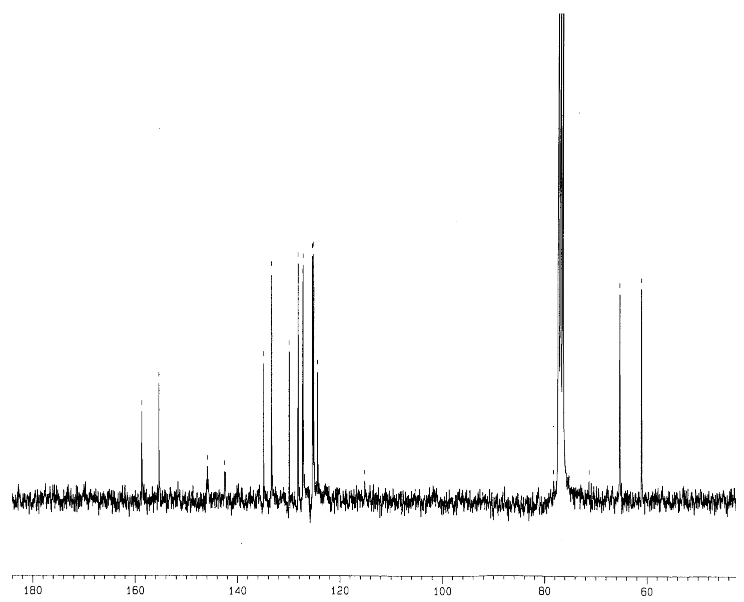

^{13}C NMR DEPT ($CDCl_3$, 75 MHz)

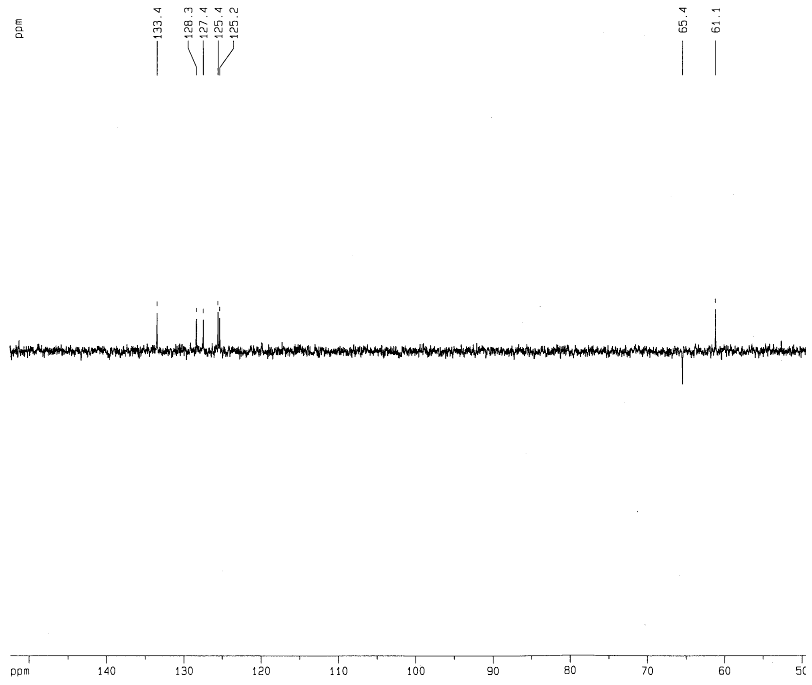

ESIMS

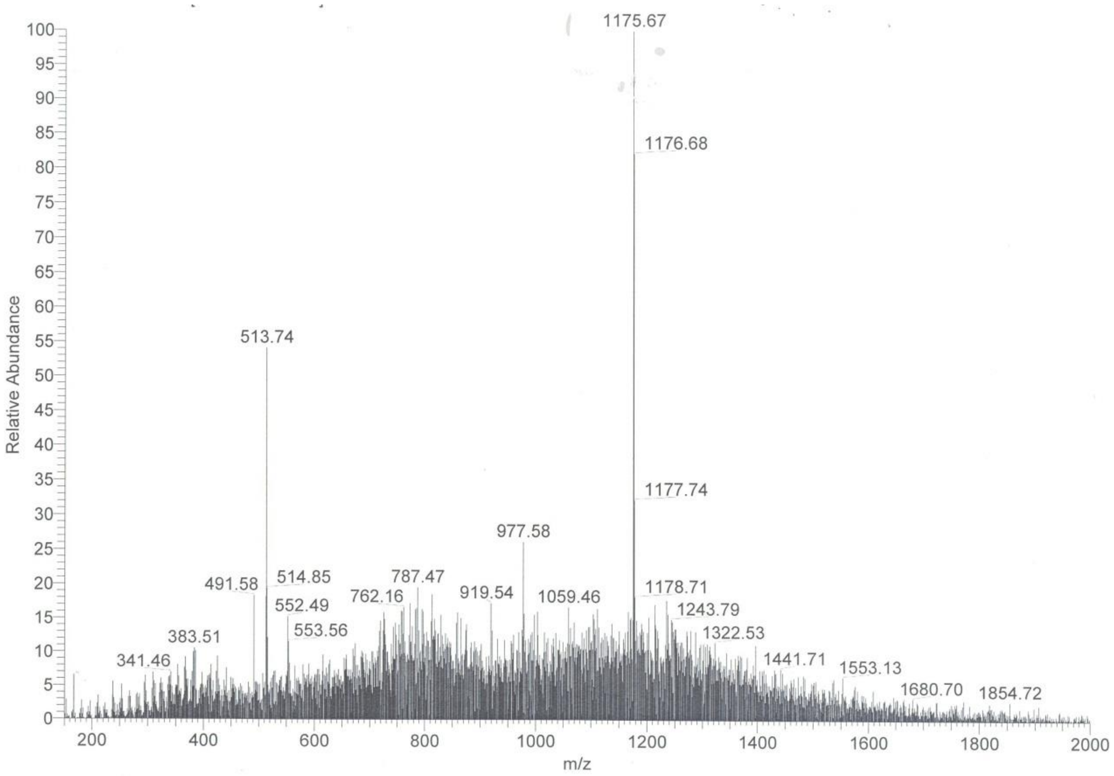

Relative Abundance
m/z
1175.67
1176.68
1177.74
513.74
977.58
491.58
514.85
552.49
553.56
762.16
787.47
919.54
1059.46
1178.71
1243.79
1322.53
1441.71
1553.13
1680.70
1854.72
383.51
341.46

Compound (*R*,*R*,*R*)-**4b**.

^{13}C NMR ($CDCl_3$, 75 MHz)

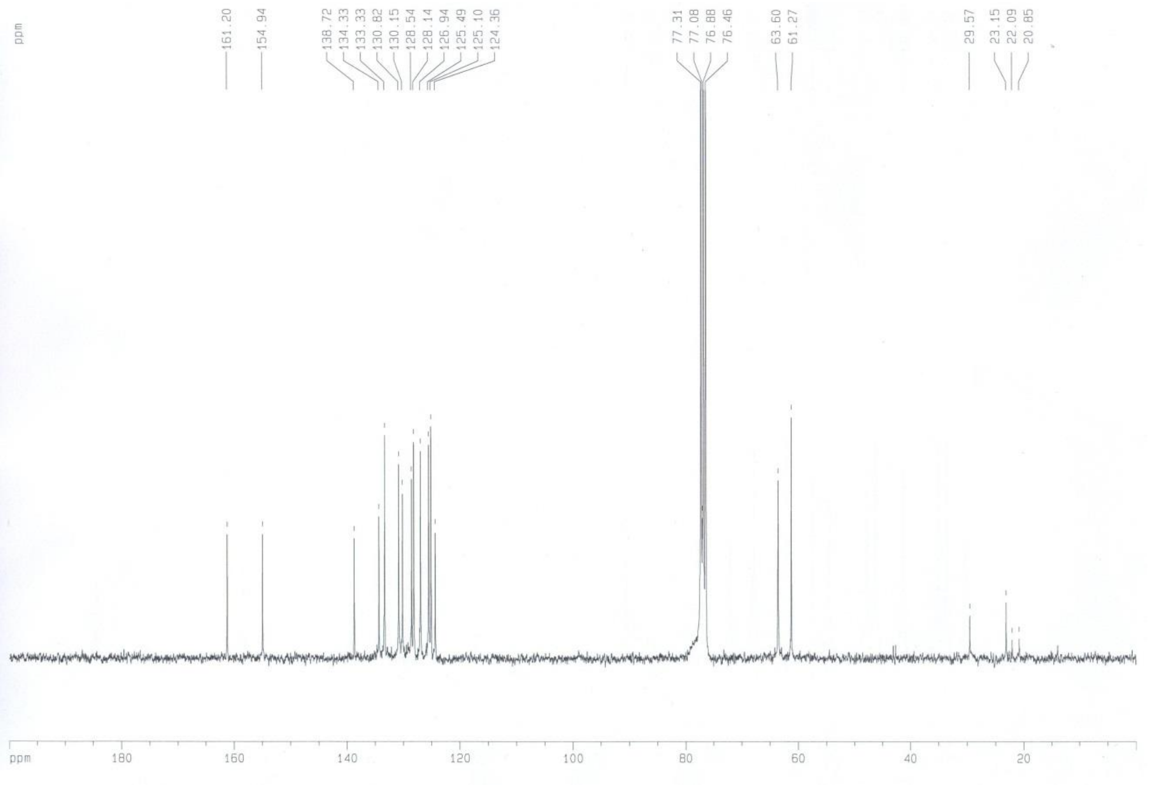

^{13}C NMR DEPT ($CDCl_3$, 75 MHz)

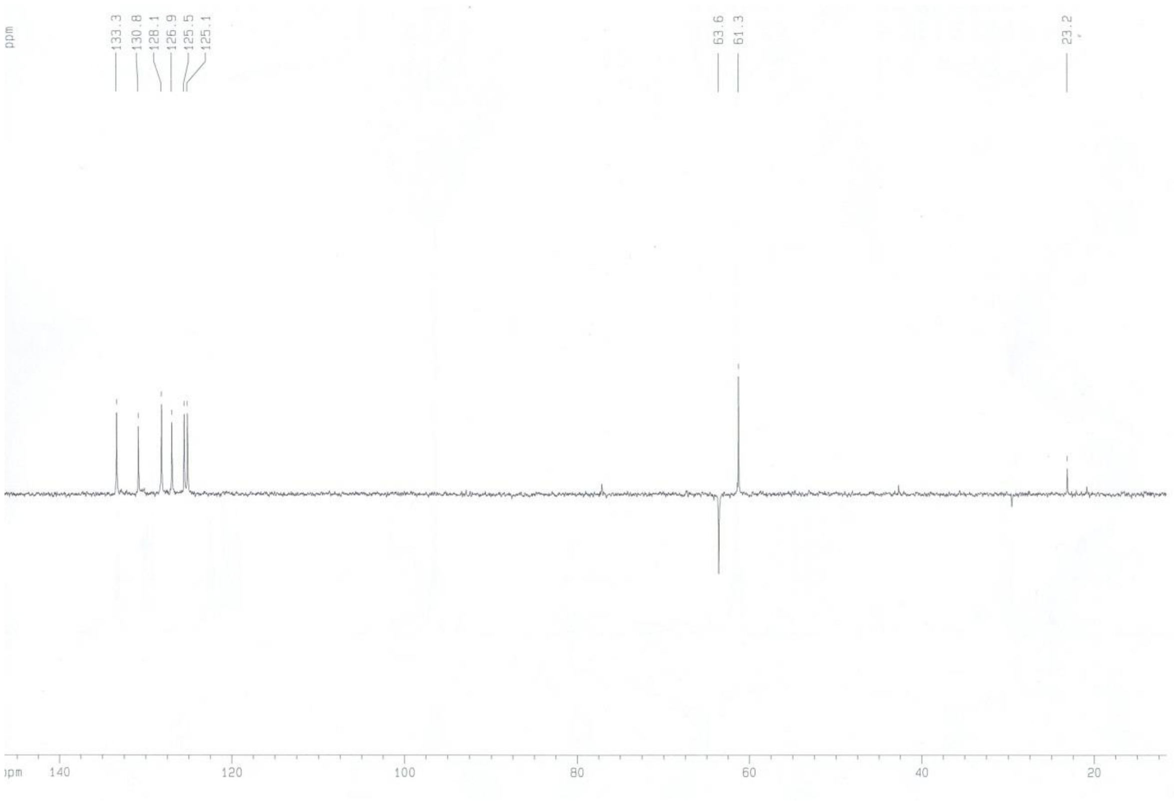

ESIMS

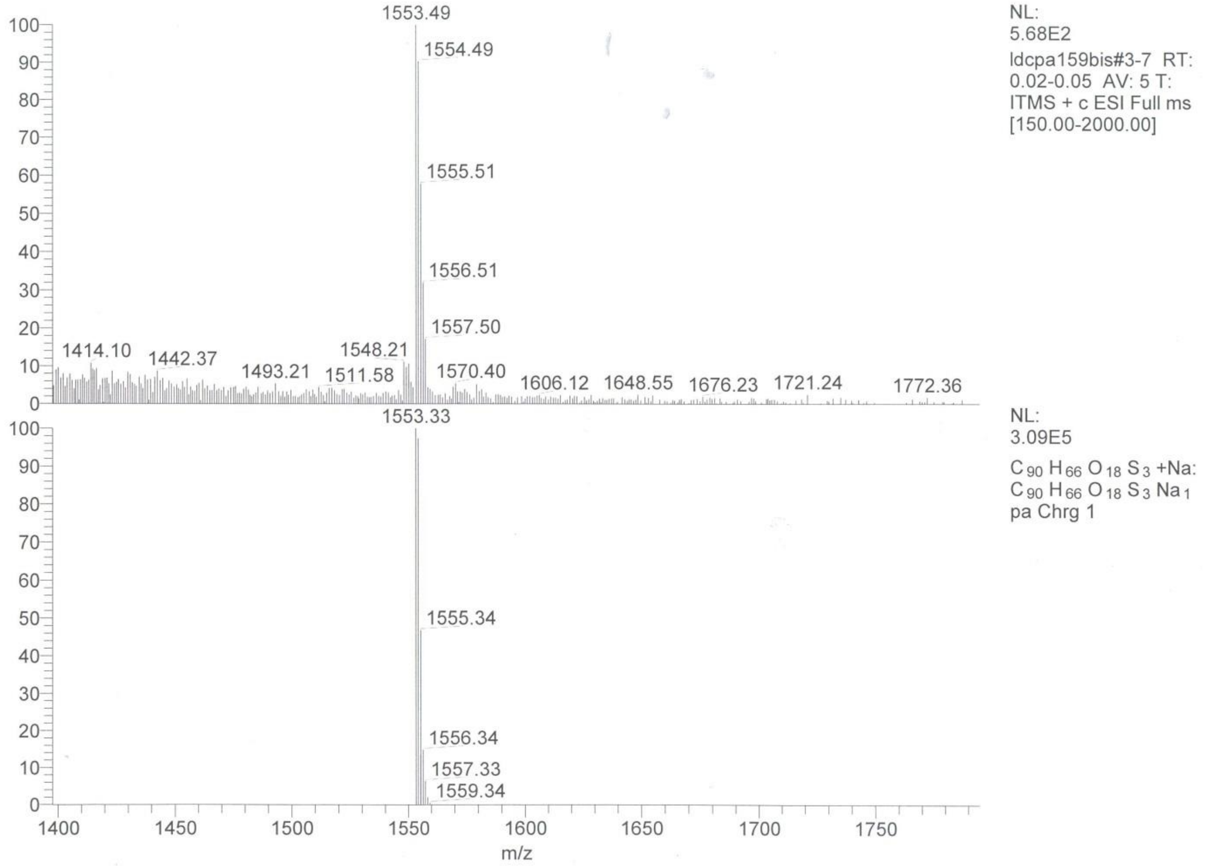

NL:
5.68E2
ldcpa159bis#3-7 RT:
0.02-0.05 AV: 5 T:
ITMS + c ESI Full ms
[150.00-2000.00]
1553.49
1554.49
1555.51
1556.51
1557.50
1414.10
1442.37
1493.21
1511.58
1548.21
1570.40
1606.12
1648.55
1676.23
1721.24
1772.36
NL:
3.09E5
C 90 H 66 O 18 S 3 +Na:
C 90 H 66 O 18 S 3 Na 1
pa Chrg 1
1553.33
1555.34
1556.34
1557.33
1559.34
m/z

Compound (*R*,*R*,*R*)-**4c**.

^{13}C NMR ($CDCl_3$, 75 MHz)

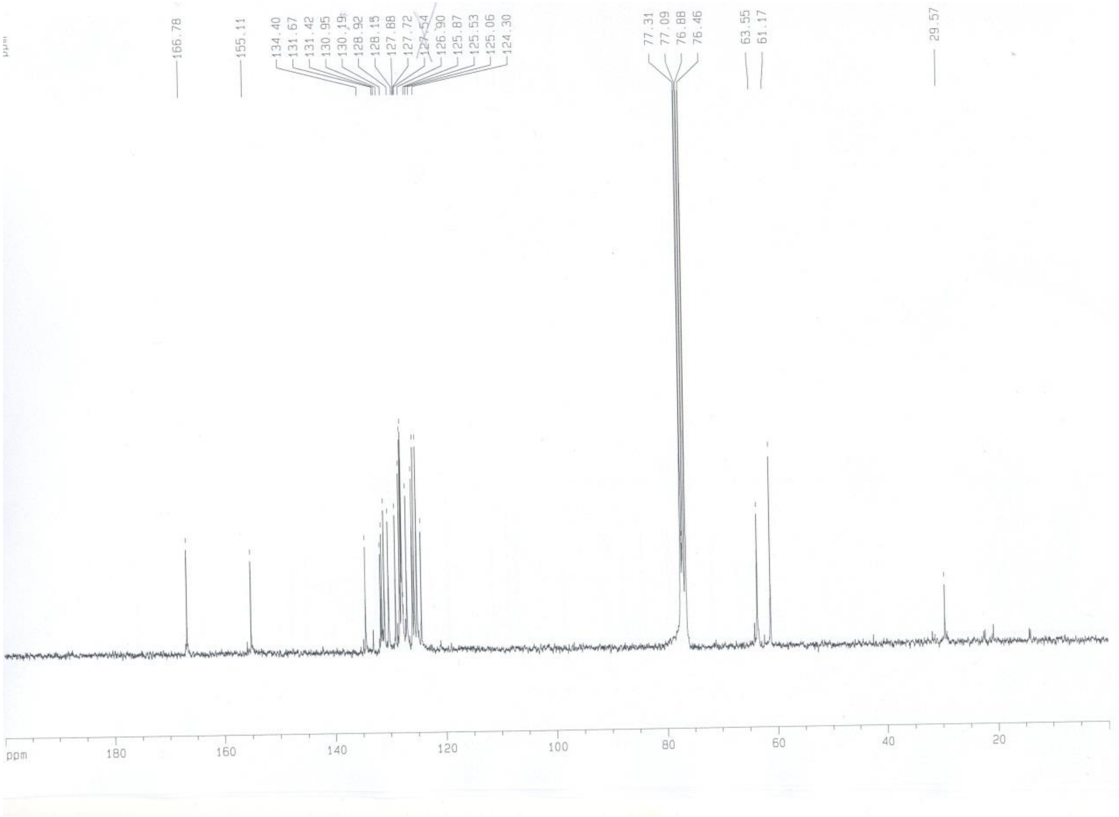

^{13}C NMR DEPT ($CDCl_3$, 75 MHz)

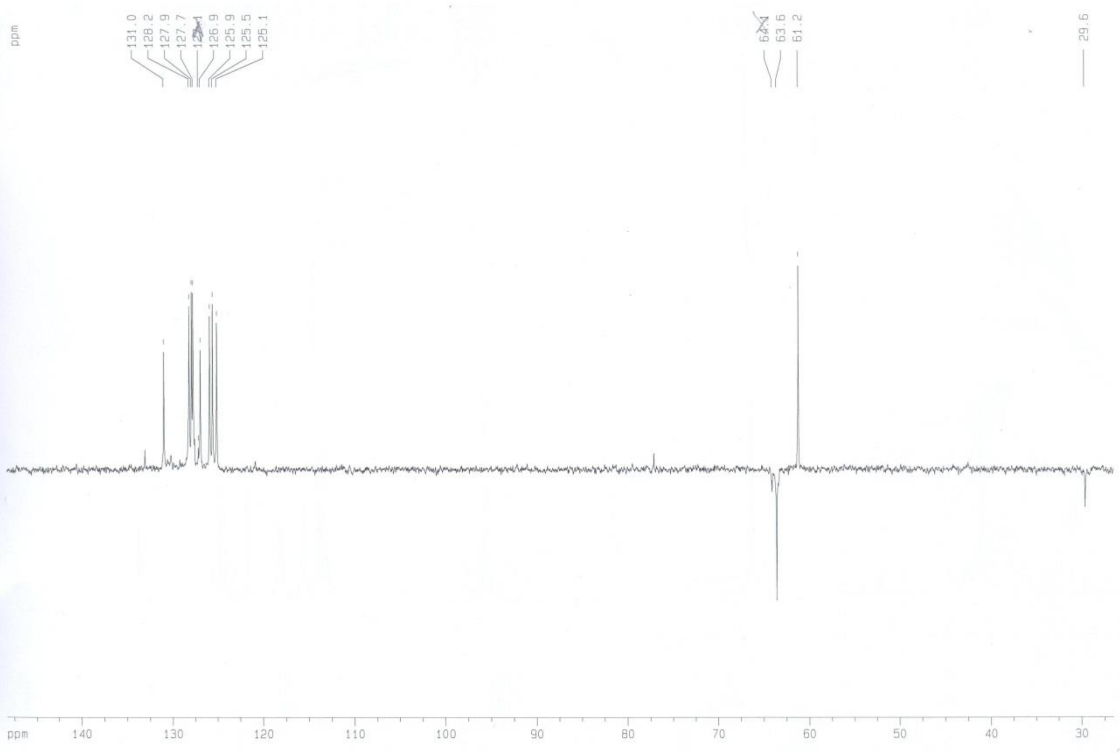

ESIMS

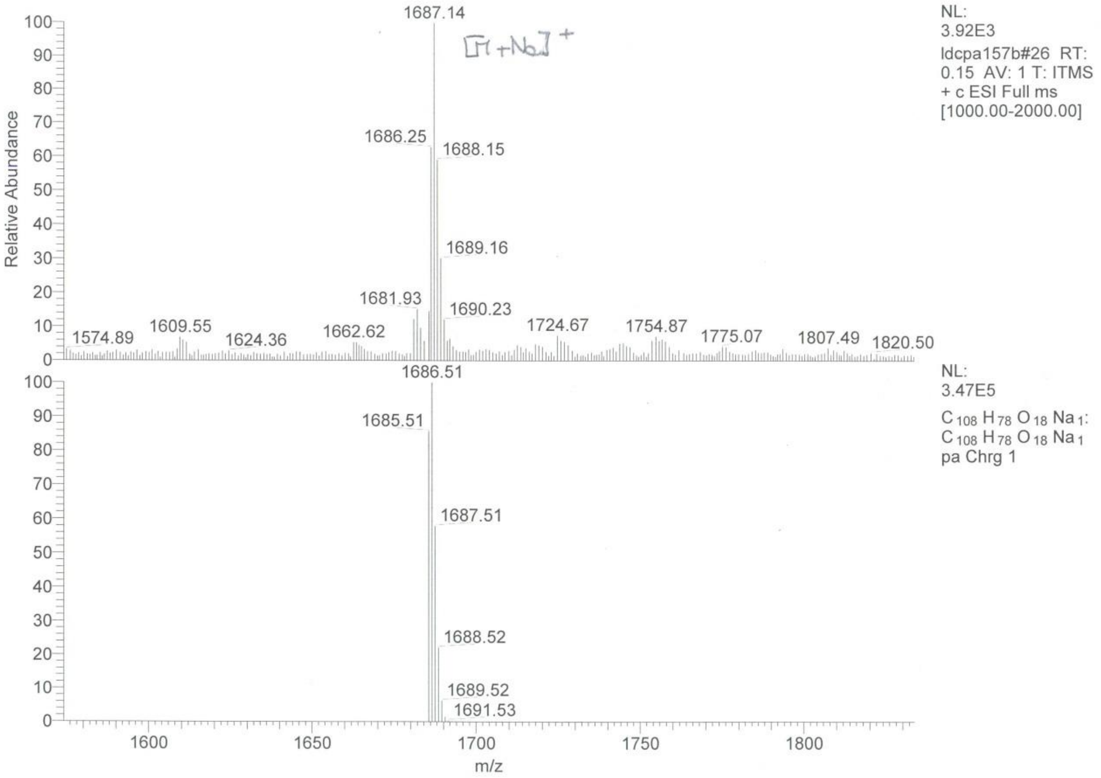
1687.14
[M+Na]+
NL:
3.92E3
ldcpa157b#26 RT:
0.15 AV: 1 T: ITMS
+ c ESI Full ms
[1000.00-2000.00]
1686.25
1688.15
1689.16
1681.93
1690.23
1574.89
1609.55
1624.36
1662.62
1724.67
1754.87
1775.07
1807.49
1820.50
Relative Abundance
1686.51
NL:
3.47E5
C 108 H 78 O 18 Na 1:
C 108 H 78 O 18 Na 1
pa Chrg 1
1685.51
1687.51
1688.52
1689.52
1691.53
m/z

Compound (*R*,*R*,*R*)-**4d**

^{13}C NMR ($CDCl_3$, 75 MHz)

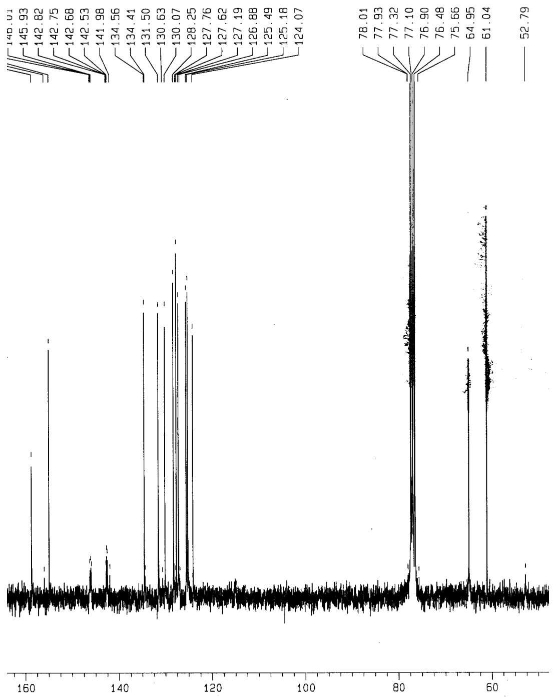

^{13}C NMR DEPT ($CDCl_3$, 75 MHz)

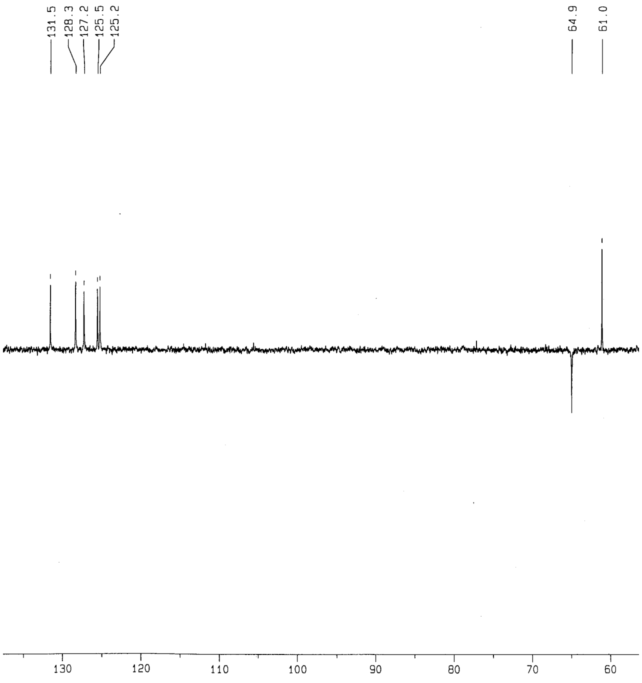

ESIMS

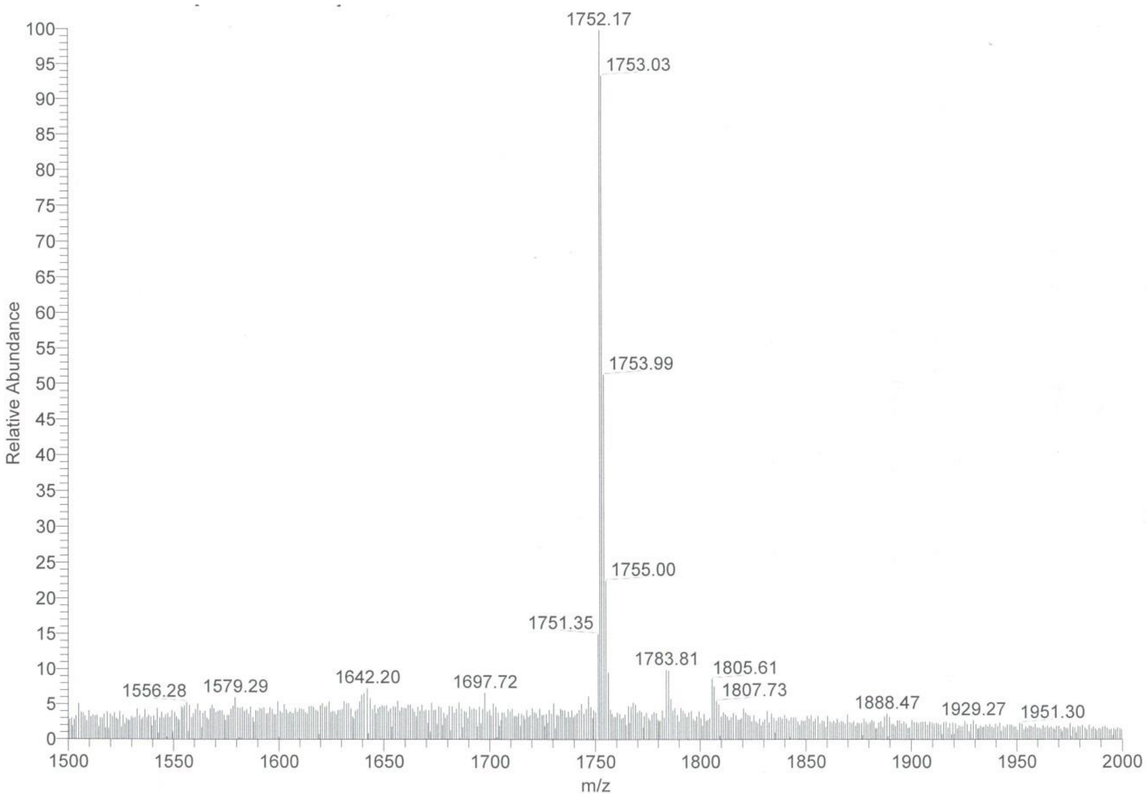

1752.17
1753.03
1753.99
1755.00
1751.35
1783.81
1805.61
1807.73
1556.28
1579.29
1642.20
1697.72
1888.47
1929.27
1951.30
Relative Abundance
m/z
100
95
90
85
80
75
70
65
60
55
50
45
40
35
30
25
20
15
10
5
0
1500
1550
1600
1650
1700
1750
1800
1850
1900
1950
2000
